# Supplementary figures and images for: The Melanoma Genomics Managing Your Risk Study randomised controlled trial: statistical analysis plan
Source: Trials. 2020 Jun 30;21:594. doi: 10.1186/s13063-020-04351-w (PMC7329549; doi:10.1186/s13063-020-04351-w)

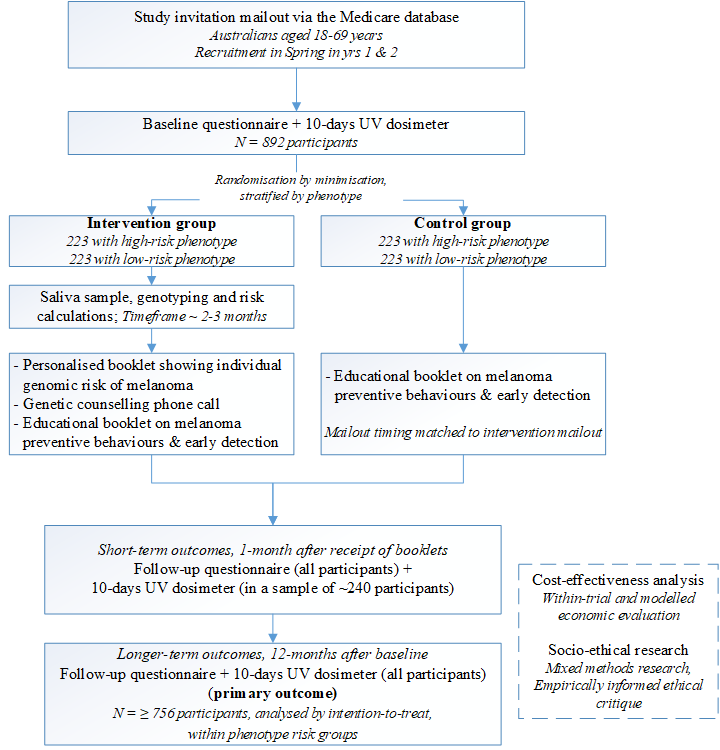

Supplement: Supplementary file 1 — Additional file 1: Supplementary Figure 1. Trial schema. [file 13063_2020_4351_MOESM1_ESM.png]

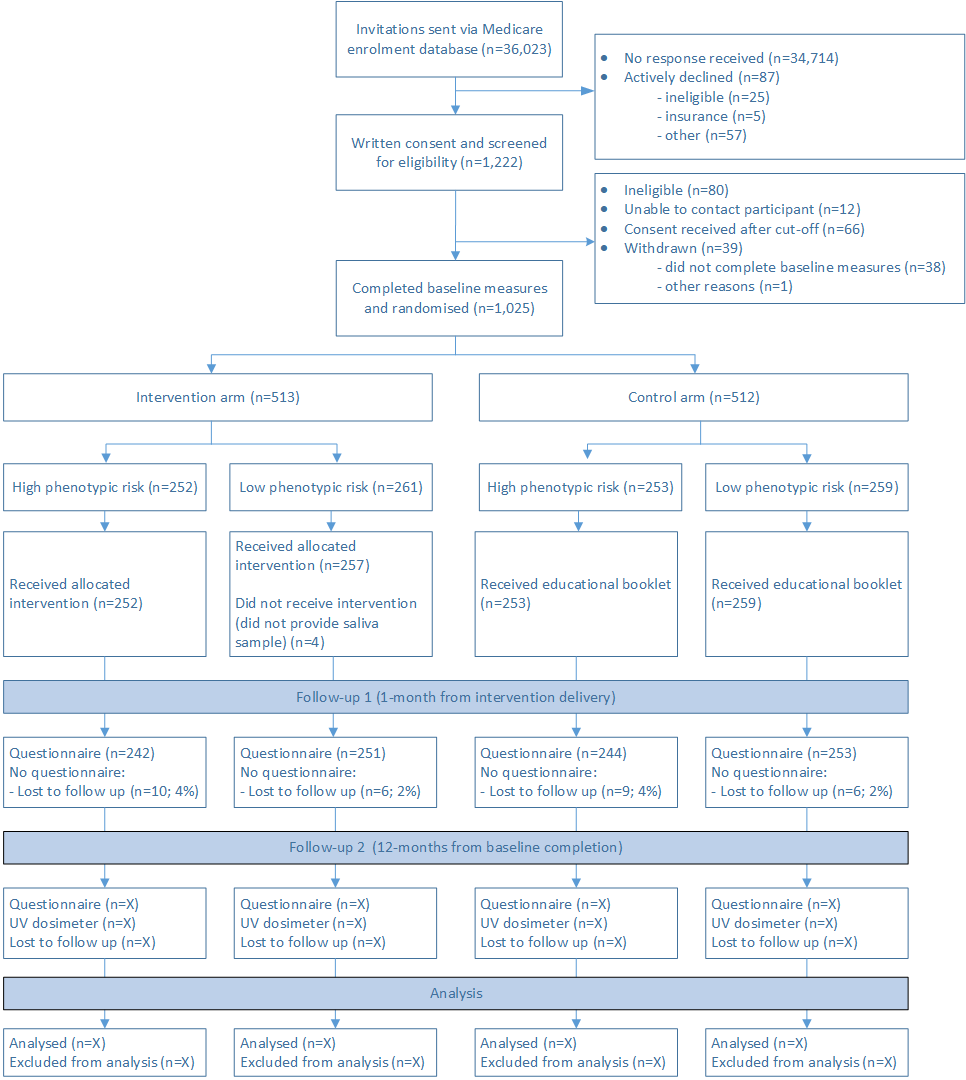

Supplement: Supplementary file 2 — Additional file 2: Supplementary Figure 2. Consolidated Standards of Reporting Trials (CONSORT) Flow Diagram. [file 13063_2020_4351_MOESM2_ESM.png]
