# Supplementary material for: The Melanoma Genomics Managing Your Risk Study randomised controlled trial: statistical analysis plan
Source: Trials. 2020 Jun 30;21:594. doi: 10.1186/s13063-020-04351-w (PMC7329549; doi:10.1186/s13063-020-04351-w)
Supplement: Supplementary file 3 — Additional file 3: Supplementary Table 1. Demographic and baseline variables. [file 13063_2020_4351_MOESM3_ESM.docx]

**Supplementary Table 1. Demographic and Baseline Variables**

| ***Information about you*** | |
| --- | --- |
| 1 | Date of birth |
| 2 | Sex |
| 3 | Highest educational attainment |
| 4 | Country of birth |
| 5 | Marital status |
| 6 | Income |
| 7 | Employment status |
| 8 | Time spent outside during a work day (paid or unpaid) |
| 9 | Family history of melanoma |
| 10 | Personal/family history of other types of skin cancer |
| 11 | Personal history of cancer |
| 12 | Family history of cancer |
| 13 | Children |
| ***Your colouring, skin type and moles*** | |
| 14 | Hair colour |
| 15 | Skin colour |
| 16 | Exposure to sunlight |
| 17 | Eye colour |
| 18 | Moles |
| ***Your sun habits and skin checks*** | |
| 19 | Skin self-examination |
| 20 | Professional skin examination |
| 21 | Advice from doctor |
| 22 | Social norms about skin-examinations |
| 23 | Skin Self-Examination Attitude Scale (SSEAS) |
| 24 | Anxiety skin examinations |
| 25 | Sun exposure |
| 26 | Sun protection habits (SPH) index |
| 27 | Sunscreen use |
| 28 | Protective behaviours - importance |
| 29 | Protective behaviours - perform |
| 30 | Sunscreen use barriers |
| 31 | Protective clothing barriers |
| 32 | Sun barriers |
| 33 | Hat |
| 34 | Social norms about sun protection |
| 35 | Sunburn |
| 36 | Intentional tanning |
| 37 | Solarium use |
| ***Your thoughts about sun habits*** | |
| 38 | Perceived effectiveness of specific sun-protection behaviours in reducing personal melanoma risk (response efficacy) |
| 39 | Vitamin D knowledge |
| 40 | Tanning (pro-tan score) |
| 41 | Discussion about sun protection and screening behaviours with family members |
| ***Your thoughts about melanoma*** | |
| 42 | Perceived control over the development of future melanomas |
| 43 | Perceived control over early detection of future melanomas |
| 44 | Confidence identifying melanoma (self-efficacy) |
| 45 | Perceived influence of genetic factors on melanoma risk (genetic determinism) |
| 46 | Melanoma related worry |
| 47 | Information seeking skin cancer |
| 48 | Information seeking genetics |
| 49 | Perceived severity of melanoma |
| 50 | Perceived risk of melanoma |
| ***Your thoughts about certain activities and behaviours*** | |
| 51 | Risk propensity risk taking |
| 52 | Risk propensity risk perception |
| 53 | Risk propensity risk attitude |
| ***Your general health*** | |
| 54 | Psychological distress and well-being using the 5-item version of the Mental Health Inventory (MHI-5) |
| 55 | Medications that may increase risk of melanoma or skin sensitivity to sunlight |
| 56 | General health |
| 57 | Importance of health |
| 58 | Confidence in completing medical forms |
| 59 | Confidence in understanding medical information |
| 60 | Private health insurance |
| 61 | Reasons for participating in the study |
